# The Immune System as a Therapeutic Target for Alzheimer’s Disease

**DOI:** 10.3390/life12091440

**Published:** 2022-09-16

**Authors:** Tarek Zieneldien, Janice Kim, Darrell Sawmiller, Chuanhai Cao

**Affiliations:** 1 Department of Pharmaceutical Science, Taneja College of Pharmacy, University of South Florida, Tampa, FL 33612, USA; 2 MegaNano BioTech, Inc., 3802 Spectrum Blvd. Suite 122, Tampa, FL 33612, USA; 3 USF-Health Byrd Alzheimer’s Institute, University of South Florida, Tampa, FL 33613, USA

**Keywords:** immunotherapy, peripheral immune system, aging, microglia, Alzheimer’s disease

## Abstract

Alzheimer’s disease (AD) is a heterogeneous neurodegenerative disorder and is the most common cause of dementia. Furthermore, aging is considered the most critical risk factor for AD. However, despite the vast amount of research and resources allocated to the understanding and development of AD treatments, setbacks have been more prominent than successes. Recent studies have shown that there is an intricate connection between the immune and central nervous systems, which can be imbalanced and thereby mediate neuroinflammation and AD. Thus, this review examines this connection and how it can be altered with AD. Recent developments in active and passive immunotherapy for AD are also discussed as well as suggestions for improving these therapies moving forward.

## 1. Introduction

AD is a neurodegenerative disorder initially described by Alois Alzheimer in 1906 [1]. Currently, AD is the most common cause of dementia, and has an advancing global prevalence without any apparent cure [2]. In general, AD is a heterogenous and multifactorial disease, with some of the major known pathological factors consisting of aggregated amyloid beta and phosphorylated tau. The accumulation of excess Aβ aggregated into toxic fibrillar deposits in the brain has been implicated in dementia and neuronal degeneration via the disruption of synaptic and neuronal function [3]. Furthermore, AD can be grouped into different subtypes based upon the pathological factors, with subjects differing in terms of gender distribution, age of onset, cognitive deterioration, and APOE genotype [4]. Due to this, AD manifests itself as a multi-domain amnestic disorder, with different patients revealing variant syndromes [4,5]. Furthermore, aging appears to be the most critical factor for AD due to its considerable repercussions on the immune system [6].

Despite our advancing knowledge of AD pathogenesis, there have been more than 200 unsuccessful clinical trials in the last decade [7]. In general, this could be attributed to recruiting subjects with a high degree of heterogeneity, which might lead to inappropriate single protein targeting in such a multifactorial disease [5,7,8]. In the present review, the effects of aging on the peripheral immune system are discussed, as well as recent advances in active and passive immunotherapies in an effort to rebalance the immune system of AD patients. Suggestions are also offered in the form of personalized medicine in an effort to find effective immunotherapies for AD treatment.

## 2. Pathological Theories of AD

Alzheimer’s disease is characterized by the accumulation of amyloid-beta (Aβ) formed by neuritic plaques and neurofibrillary tangles (NFTs) [9]. The two components of neuropathological changes associated with AD include positive lesions due to the accumulation of deposits in the brain and negative lesions due to losses by atrophy resulting from neural and synaptic loss [9]. The hallmark proteinopathies for AD include Aβ and pathologic tau which serve as potential biomarkers for onset of AD [10]. Studies have shown abnormal amyloid deposition can lead to rapid decline of cognition, progressive atrophy, and hypometabolism [10].

**Figure 1:**
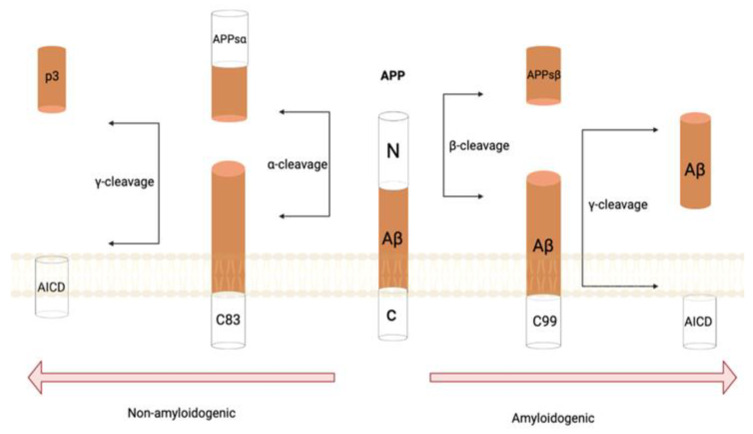
APP amyloidogenic and non-amyloidogenic processing pathways. In the nonamyloidogenic pathway, APP is cleaved by α-secretase to produce a soluble amino-terminal of the amyloid precursor protein, as well as a C-terminal fragment (C83) that can subsequently be cleaved by γ-secretase to produce the p3 extracellular fragment and amyloid precursor protein intracellular domain (AICD). In the amyloidogenic pathway, β-secretase cleaves APP to generate the soluble amyloid precursor protein β- and a C-terminal fragment (C99). Then, the cleavage of C99 by γ-secretase leads to the release of amyloid-β and AICD.

Amyloid precursor protein (APP) is a type 1 membrane protein which plays a significant role in the development of AD with the proteolytic activity of β- and γ-secretase complex [11]. The two byproducts of APP metabolism that are generated in neurotoxic amyloid plaques consists of abnormally folded Aβ40 and Aβ42 [11]. An imbalance between the production and clearance of these alternate forms can initiate AD pathogenesis. Aβ42 experiences conformational changes, making it more prone to aggregation into oligomers due to the increased hydrophobic nature in the C-terminus, furthering the formation of fibrils, plaques, and phosphorylated tau. This results in neuritic dystrophy and furthers the spread of neurodegeneration [12]. The oligomeric Aβ42 is found in abundance around plaques, making oligomers the causative agent for neurodegeneration while also inducing tau hyperphosphorylation [13]. It has been found in animal models that Aβ oligomers impair memory, inhibit long-term potentiation, and decrease synapse density [13]. As such, an approach to AD treatment has been to slow Aβ peptide formation in the brain via the manipulation of γ- and β-secretases (Figure 1) [14]. In general, immunotherapy targeting Aβ has been prominently used in AD because the accumulation of Aβ within the brain has been noted to be an early trigger [15].

Tau pathology is another contributing factor in AD pathogenesis. The essential functions of tau proteins are to promote the interaction and stability of microtubules and tubulin in the neural network [16]. In AD, however, tau mutations can occur inhibiting these vital functions [17]. Seemingly interconnected in the formation of neuritic plaques are microglia, Aβ, and tau, where the reaction between Aβ and microglia results in tau accumulation [18]. The main forms of tau that contribute to AD progression are misfolded, aggregated, and hyperphosphorylated forms which spread throughout the brain in a prion-like manner, inducing further aggregation through formation of paired helical filaments [19]. The hyperphosphorylated tau proteins create neurofibrillary tangles that accumulate within axons and dendrites resulting in neuronal loss [20]. Over time, the toxicity of tau is enhanced due to the alterations in the kinases or phosphatases that target tau, thereby suppressing and silencing many neurons [21].

Genetics can influence the risk of developing AD, as seen with the association of the apolipoprotein E (APOE) genotype with AD [22]. The variants of the APOE gene include ε2, ε3, and ε4 alleles, with the APOE ε4 isoform being a crucial genetic risk factor having significant detrimental effects in AD development [23]. APOE ε4 has been associated with a lower age of onset and elevated prevalence of AD, with the clinical onset age being 68 years in APOE ε4 homozygotes and 76 years in APOE ε4 heterozygotes, compared to 84 years of age in non-ε4 carriers [24,25]. This increased risk is associated with the APOE ε4 allele inhibiting Aβ clearance and advancing Aβ aggregation [26].

## 3. Effects of Aging on AD

Aging is typically considered the most critical risk factor for AD and has extensive repercussions for the peripheral tissues and immune system [27]. Aging has been determined to alter the constituents of innate immunity extending from the behavior of dendritic cells, natural killer cells, monocytes, and neutrophils, as well as the expression of the signaling molecules [28]. This is crucial because innate immunity offers vast host protection via the detection of pathogen-associated molecular patterns (PAMPs) and the activation of signaling pathways that lead to the expeditious release of chemokines and cytokines, which are pivotal soluble molecules that serve as immune effectors [29]. Subsequently, inflammation is also witnessed with aging, and is distinguished by functional deterioration and incessant low-grade inflammation in aging individuals [30]. The intracellular multicomponent sensors and receptors that allow for the release of the eminent pro-inflammatory cytokines IL-18 and IL-1β are inflammasomes [31,32]. Consequently, the distinct inflammasome expression of gene modules in geriatric patients has been correlated with arterial stiffness, oxidative stress, high blood pressure, and metabolic impairment, thereby leading to reduced patient life spans [33]. Correspondingly, the elevated levels of IL-18 circulating due to increased age show a significant decrease in mice that lack the Asc inflammasome adaptor or the Nlrp3 inflammasome [34]. Nonetheless, a plethora of proinflammatory factors and cytokines have been correlated with age-related cognitive and physical deterioration, and some such as IL-6 have been utilized as markers of dysfunctional inflammatory responses [35].

Aging also correlates with elevated cell transcriptional fluctuations and volatility, inclusive of hematopoietic cells [36,37,38]. Proportionately, older cells from the peripheral immune system encompass epigenetic modifications that are eminently heterogenous when comparing differing individuals and even different cells, as shown by profiling of the single-cell chromatin modifications [38]. Furthermore, considerable variances in the cell populations that arbitrate adaptive immunity have been witnessed in a variety of research studies, with a reduction in circulating B cells and naïve T cells being common, while T cells that are terminally differentiated seem to be found in elevated levels in geriatric subjects [28,39]. In general, this is attributed to the thymic involution that occurs with age and is mediated by the inflammasome Nlrp3, leading to a deficiency in T-cell homeostasis [34]. Furthermore, aging has detrimental effects on naïve T cells since they tend to retain a more limited range of T-cell receptor repertoires, leading to debilitated virtual memory phenotypes, which are consorted with extensive epigenetic alterations [38,39,40,41]. Consequently, CD4+ and CD8+ T cells tend to exhibit characteristics reminiscent of cellular senescence as they become more aged [29].

Aging results in an amassment of senescent cells since they enter a long-standing scheme of cell-cycle arrest that is elicited by a plethora of stressors [29]. Recently, research has demonstrated an intricate mechanism that is fundamental to cell biogenesis, as well as elucidated their actions in distinct pathology and physiology [42,43]. In host immunity, the engagement of senescent cells is intertwined to their attained capability of secreting pro-inflammatory cytokines, known as the senescence-associated secretory phenotype [44]. The senescence-associated secretory phenotype is induced principally by oncogenic stress, NF-κB in response to damaged DNA, and developmental cues, which initiates the IL-6, IL-8, IL1β, and TNF-α transcription [45].

Consequent to inducing a pro-inflammatory phenotype, aging also diminishes molecules that are necessary for brain rejuvenation [46]. To illustrate, when old mice collectively shared their circulation with younger mice via parabiosis, there appeared to be transmission of aging phenotypes to the brain and peripheral tissues of youthful mice, with the converse scenario also taking place [47]. In fact, B2M and CCL11 immune factors have also been demonstrated to adversely influence memory and neurogenesis when enriched in the plasma of geriatric individuals [29,47]. However, the complementing of growth and differentiation factor 11 (GDF-11) to older mice has been demonstrated to benefit the heart and boost neurogenesis [48]. Nevertheless, GDF-11 activity and expression is a contentious topic and subsequent research is crucial to corroborate its activity in the rejuvenation of the host. However, an almost full-fledged restoration of neuronal and synaptic proteins has been observed in elderly APP-expressing mice subsequent to exposing the mice to circulating juvenile blood or after obtaining a plasma transfusion [49]. This resulted in enhanced associative and working memory after intravenous administration of the plasma of younger mice when there were no alterations in the amyloid load [49]. Consequently, a profound exploration into factors that contribute to brain rejuvenation has allowed for the recognition of tissue inhibitor of metalloproteases 2 (TIMP2), which is found in the umbilical cord blood at greater amounts [50]. TIMP2 is critical since it boosts neuronal plasticity in the brain via the possible modulation of the extracellular matrix, but it also influences hippocampal synaptic plasticity via the systemic circulation [29].

Aβ aggregation and deposition seems to increase with age, which could be associated with the pathological aggregation found in AD [51]. In fact, it has been elucidated that exceptionally insoluble proteins from older *Caenorhabditis elegans* or mice brains could commence Aβ aggregation in vitro, but this was not seen when younger subjects were utilized [51]. Furthermore, insoluble Aβ has also been shown to lead to neuronal stress, thereby increasing Tau-expression and phosphorylation, while also leading to microglia induced inflammation [52]. Although tau pathology seems to be more strongly correlated with AD-related cognitive dysfunction than Aβ, the Aβ cascade is considered an earlier process, and early intervention preventing Aβ aggregation could help immune dysfunction [53].

### 3.1. Effects of the Peripheral Immune Cells in Aging-Related Brain Homeostasis

In the parenchyma, microglial cells can be found, and a limited but significant quantity of NK cells, B cells, T cells, and dendritic cells roam to the choroid plexus and meninges to populate them [54]. Various studies have determined that CD4+ T cells have an imperative role in preserving naïve mice behavioral and cognitive capacities [55,56]. Both T_H_1 (IFN-γ producing) and T_H_2 (IL-4-producing) CD4+ T cells populate the meninges in steady-state conditions [57]. IFNγ assists crucial neuronal circuits that are utilized for social behaviors and IL-4 eases learning via the regulation of meningeal dendritic cells and the stimulation in astrocytes of BDNF expression [57,58]. As such, T cells and their secreted cytokines have crucial roles in maintaining homeostatic brain processes [29]. Nonetheless, aged choroid plexus in both mice and humans show strong IFN-I signaling, which has been demonstrated to counter the function of IFNγ, hinder hippocampal neurogenesis and cognitive functions, as well as disrupt monocyte infiltration [59]. Therefore, aging potentially disrupts the balance of brain-homing peripheral immune cells, thereby negatively impacting crucial functions of the CNS [59].

The choroid plexus is an epithelial tissue found within the brain’s ventricles [60]. The choroid plexus forms the blood–CSF barrier and has a crucial role in preserving homeostasis of the brain by excreting neurotrophic factors into the CSF, participating in Aβ clearance, and trafficking leukocytes [60]. When observing naïve mice, it has been found that the stroma of the choroid plexus contain greater than 50% of CD4+ and CD8+ T cells in the brain [29]. The majority of these T cells embody effector memory phenotypes—inclusive of T_H_1, T_H_2, and T_regs_—and have the ability of recognizing antigens in the CNS [61]. Nonetheless, aged choroid plexus demonstrates a contorted T_H_1 to T_H_2 balance ratio, leading to elevated expression of the CCL11 chemokine, reduced permeability to leukocytes due to IL-4 and IFNγ differential impacts on the epithelial cells of the choroid plexus, and hindered cognition [62]. Type I interferon (IFN-I) is a considerable innate immune cytokine frequently implicated in host defense and autoimmune circumstances [29]. Furthermore, the aged choroid plexus of both mice and human subjects exhibit compelling IFN-I signaling, which has been revealed to counterbalance the actions of IFNγ, disturb monocyte infiltration, and hinder neurogenesis of the hippocampus and cognitive function [61]. Due to this, aging has been determined to disturb the balance of the peripheral immune cells in the brain, which have detrimental effects on the function of the CNS [63].

### 3.2. The Innate Immune System

In AD pathology, observations into the brain can elucidate microglial differentiation into a novel form that is correlated with neurodegenerative diseases, encompassing modified molecular expression profiles, as well as constrained phagocytic capacities [64]. Nonetheless, the definite source of the amoeboid senile plaque surrounding myeloid cells has been a topic of much discussion, due to the strenuous task of characterizing locally activated microglial cells with those infiltrating myeloid cells [29]. However, contemporary studies have indicated that peripheral macrophages could retain an individualized transcriptional and functional identity in the CNS while engrafting the brain [65]. Furthermore, infiltration of the peripheral myeloid cells has also been indicated to engage in the clearance of Aβ [66].

Unusual variants in the triggering receptor expressed on myeloid cells 2 (TREM2) have also been observed to elevate AD development by at least twofold [29]. Consequently, TREM2-dependent phenotypes in mice model studies have shown that the phenotypes can elucidate the role of microglia and TREM2 in CNS injury, and thereby shed light on AD pathogenic neuroinflammation [67]. Moreover, other than the upregulation within the CNS, TREM2 mRNA and protein also show elevated expression in the peripheral leukocytes of subjects with AD, which has been associated with atrophy of the hippocampus and cognitive deficits [68,69]. Nonetheless, it is uncertain whether increased expression of peripheral TREM2 indeed has an operative repercussion or if it resonates the continuous systemic inflammation observed in AD patients.

Neutrophils are the myeloid cells with the most abundance in the peripheral blood of humans [29]. Neutrophils have crucial roles in the innate immune system and are present in the brain parenchyma of 5xFAD and 3xTg-AD mice [70]. In the brain parenchyma of these mice, it was noted that neutrophils seemed to boost cognitive decline, amyloid plaques, and tau tangles [70]. However, further research revealed that treating 3xTg-AD mice for 10 months with TNF-α modulator compounds yielded elevated infiltration of the brain by neutrophils, which concurred with decreased amyloid and tau pathologies and enhanced memory [71]. As such, further research is necessary to elucidate the operative characteristics of infiltrating neutrophils in AD pathogenesis.

### 3.3. The Adaptive Immune System

The antibody production by B cells to amyloid beta has been extensively studied over the past two decades [72]. In fact, immortalized B cells were initially discovered to secrete antibodies with specificity towards Aβ peptides in the peripheral blood of subjects with AD [72]. As of now, anti-Aβ antibodies have been found to circulate at differing levels in human blood, regardless of AD diagnosis [73,74]. This led to the consideration of B-cell mediated immune responses as a therapeutic strategy, since studies have shown that Aβ immunization has the potential of preventing the advancement of amyloid plaques, as well as gliosis and neuritic dystrophy in PDAPP mice [75]. Nevertheless, as witnessed in the AN1792 clinical human trials, the development of encephalitis in approximately 6% of the subjects led to termination of the trials [76]. In this case, the encephalitis was potentially traced to the T_H_1 response stimulation via active immunization [76]. Consequential animal model research also indicated that antibody clones that are Aβ -specific could ameliorate AD progression without the engagement of T cells, thereby encouraging passive immunotherapies in the clinical trials of AD subjects [77]. Nonetheless, passive immunotherapy has shown varied results, and a plethora of clinical trials are still continuing [78,79].

Other than specific humoral responses to proteins correlated with the pathology of AD, the repertoire of peripheral immunoglobulin appears to be abnormally regulated [29]. To illustrate, natural antibodies that are released by B cells devoid of exogenous invigoration are copious in human sera, commonly self- or poly-reactive, and effectuate crucial activity in the removing of cellular waste and targeting of pathogens [80]. Consequently, the levels of natural IgG recognizing self-antigens has been shown to be affected by disease and age [81]. When observing other neurodegenerative disorders, a significant decrease in autoantibodies was detected in AD, multiple sclerosis, and Parkinson’s disease (PD) patient sera [81]. However, the inciting factor and operative significance of this reduction has not yet been elucidated. Nonetheless, antibody panels have been utilized as biomarkers, and some studies have demonstrated the ability to differentiate AD and MCI patients from age-matched controls [82].

Subsequent studies have also provided additional intuitiveness on immunoglobulin’s role in AD [83]. To illustrate, elevated levels of mice IgGs were observed in correlation with microglia in 5xFAD mice brains [29]. Deficient in Aβ specificity, the proteins were revealed to interact with the Fc receptor of microglia, leading to the activation of the signaling pathway and triggering the phagocytosis of Aβ, which thereby results in reduced plaque loads [29]. However, even though the study conducted by Marsh et al. did not examine the infiltrating IgG’s antigen specificities, the defensive effects of mice IgG are evocative of the favorable effect yielded by intravenous immunoglobulin administration [83]. However, there have also been unfavorable reports documenting low-dose intravenous immunoglobulin administration in human clinical trials. In a Phase III trial conducted by Relkin et al., there were no beneficial effects observed for the two administered doses (0.2 and 0.4 g/kg every 2 weeks for 18 months), although the subjects with mild- to moderate-AD showed good tolerability of treatment [84]. Even more, there were no difference between the placebo and intravenous immunoglobulin treated subjects in terms of the rate of occurrence of amyloid-related imaging abnormalities (ex., microhemorrhages or brain edema) [84]. Nonetheless, the definitive subsets of IgGs that deliberate augmented neuroprotection warrant further research.

In terms of T cells, the brains of AD patients post-mortem have been found to have CD4+ and CD8+ T cells, periodically near microglia and neuritic plaques [85,86]. Likewise, elevated levels of T cells have been observed to infiltrate the parenchyma of various APP transgenic mice [87]. To illustrate, Browne et al. demonstrated a significant portion of infiltrating T cells with ability to generate IL-17 and IFNγ in APP/PS1 brains [87]. Nevertheless, other studies have revealed that infiltrating T cells continuously demonstrate an inactivated phenotype with decreased IFNγ producing abilities and a scarcity of local proliferation [88]. Furthermore—in Tg2576, APP/PS1, and ArcAβ models of AD—they did not appear to co-localize with the amyloid plaques [88].

Due to the distinct and critical activities of T helper subsets in various diseases, it would be crucial to disseminate how varying T_H_ cell populations explicitly modulate AD. In APP/PS1 mice models, adoptive transfer of Aβ-specific T_H_1 cells—although not T_H_2 or T_H_17 cells—led to exacerbated AD pathology, excessive microglial activation, and impaired cognitive function [87]. The plaque clearing and pathogenic actions of T_H_1 cells were elucidated when after Aβ vaccination, infiltration of Aβ-specific IFNγ producing T cells occurred in the brains of J20 mice, thereby clearing plaques, but causing meningoencephalitis, mirroring the pathogenicity of AN1792 [89]. Nevertheless, direct cerebrospinal injections of Aβ-specific T_H_1 cells elevated neurogenesis and amyloid plaque clearance in the absence of autoimmunity, suggesting an additional impact of peripheral T_H_1 cells in the APP/PS1 mice models [90]. In addition, the adoptive transfer of Aβ-specific T_H_2 cells, while lacking evidence of brain infiltration, enhanced the working memory of APP/PS1 mice, in addition to minimized vascular amyloidosis and systemic inflammation [91]. As such, Aβ-specific T_H_1 and T_H_2 cells seem to alter the pathology of Aβ in a plethora of differing ways.

### 3.4. Central-Peripheral Neuroimmune Crosstalk in AD

In vivo assessments of the CNS immune dysfunction in humans have been methodologically limited [92]. However, numerous studies have demonstrated that upregulated CSF pro-inflammatory markers manifest in early AD, thereby leading to an inflammatory response in the CNS [93,94,95]. In general, pathological and clinical correlations with the inflammatory response are varied and marker contingent, accentuating the fact that granular assessment of particular pathways are crucial at each stage of AD severity [92]. To illustrate, various studies have demonstrated that elevated levels of CSF chemokine (C-C motif) ligand 2 (CCL2) envision expedited clinical deterioration in AD, particularly during pre-dementia stages [94,95]. On the other hand, other studies have indicated that elevated levels of soluble triggering receptor expressed on myeloid cells 2 (TREM2) and convergence of various anti-inflammatory and pro-inflammatory factors in CSF are predictive of a more gradual clinical deterioration in AD [93,96]. In fact, utilizing the National Institute on Aging—Alzheimer’s Association 2011 criteria allowed Meyer et al. to stratify subjects on the basis of preclinical biomarker stages, thereby leading to the discovery that CSF inflammation levels were decreased in stage 1 when compared to stage 0, but considerably higher in stage 2 [97]. The four stages that participants were allocated into were defined as stage 0 (no abnormality), stage 1 (reduced amyloid-β_1-42_), stage 2 (elevated total-tau and reduced amyloid-β_1-42_), and “suspected non-AD pathology” [92]. This unanticipated result suggests that immune marker activity could potentially diminish along with the earliest emergence of amyloid-β plaque pathology [97]. These findings highlight the importance of appropriately characterizing the extent of AD-related pathology in the comparison of clinical groups. 

Furthermore, peripheral macrophages are considerable innate immune cells that have the potential of infiltrating the CNS in neuroinflammation [92]. Nonetheless, the extent of the roles that macrophages play in infiltration in relation with AD has not been fully elucidated. There are studies that have proposed that peripheral myeloid cells could infiltrate brain tissue, thereby mitigating the deposition of Aβ and cognitive impairments in murine models of AD [98,99,100]. Nevertheless, the interchange of brain-resident myeloid cells that encompass peripheral monocytes did not alter the load of Aβ in APP/PS1 and APP23 mice models of β-amyloidosis [101,102]. Subsequently, natural killer cells also have been shown to infiltrate the brain in APP/PS1 mice pathological models [103]. In general, the mechanisms utilized by peripheral innate immune cells could be directly related to the AD-related BBB dysfunction, since these BBB alterations could potentially stimulate the infiltration via the deterioration of the cellular and molecular constituents of the pericyte, endothelial, and capillary walls [104]. In spite of this, a critical impediment in the success of passive immunotherapy has been the inadequate entry of antibodies into the CNS. In fact, it has been estimated that approximately 0.1% of peripherally administered antibodies can access the brain due to the BBB, thereby hindering the drug availability inside of the brain [105,106].

Furthermore, the crosstalk between the peripheral immune system and central immune system is limited due to the scarcity of clinical studies that directly investigate their interchanges, but the current extent of evidence for correlations between acute systemic health scenarios, peripheral inflammation, and CNS-related reactions heavily advocates for the occurrence of communication between both systems [92]. However, the timing and directionality of the implicated pathways remains to be elucidated to be effectively used in therapeutic modification. Nonetheless, concurrent measurements and comparisons between peripheral inflammation and CNS pathology propose that the levels of the majority of analytes are modestly associated between the compartments [107]. AD pathology biomarkers have also been correlated with unique inflammatory signages in CSF and blood, allowing for enhanced accuracy in the classification of AD pathology [108,109]. The differing inflammatory signatures and profiles advocate for independent and/or joint input of the central and peripheral immune systems in relation to AD, illustrating that the peripheral inflammatory microenvironment is improbable to merely be a downstream secondary effect of CNS dysfunction [92].

## 4. Risk Profile of AD Patients

Universal therapeutics that focus on AD as a homogeneous disease tend to not properly assess the risk profile of the patients receiving the therapeutics [4]. Geriatric individuals with higher education levels seem to have a decreased prevalence of dementia in comparison to geriatric individuals with no formal education [110]. In this case, cognitive activity was proposed to reduce the risk of cognitive deterioration via the elevation of the cognitive reserve [110]. Consequently, individuals with genetic variants—such as TREM2 R47H or APOE ε4 allele—have also been proposed to be at elevated risk relative to an individual that lacks these variants, even though they may not fall victim to AD until decades later, or even at all [111,112]. Therefore, there are certain well-documented risks for AD—including low education, cerebrovascular risk factors, dyslipidemia, APOE ε4 allele, and head trauma, to name a few—that should be taken into account when assessing the risk profile of AD patients [113].

Consequently, vaccines produced should also take into account the risk profile of the patients. To illustrate, preventative vaccines, which target younger members of the population, have a higher and longer antibody response with adjuvant included since they would have enhanced tolerance to the vaccine induced T-cell responses [114,115]. On the other hand, therapeutic vaccines which target AD patients should induce a rapid antibody response while still maintaining low and durable antibody titers, with the aim of limiting inflammatory responses. In this case, adjuvant should not be included if possible [116]. This would allow for less adverse effects in patients with less tolerance [117]. Even more, in terms of AD, vaccination against pathogens differs from vaccination against self-peptides such as Aβ. In this case, the immune tolerance, which is defined by failing to react to self-antigens, is an imperative characteristic of the immune system [118]. Several mechanisms are employed in the avoidance of autoimmunity—such as regulatory T-cell suppression, clonal deletion of elevated affinity autoreactive lymphocytes, and induced insensitivity in matured lymphocytes, among others [118]. In β-amyloid vaccines, there could be two opposite risks, with the vaccines not being able to transgress tolerance leading to non-immunogenic effects, with the opposite effect being that the vaccine could result in a severe autoimmune reaction [118,119]. Furthermore, current and future therapeutics should consider that AD patients have been revealed to have blood–brain barrier (BBB) damage [120]. Along with the age-dependent decline in hippocampal BBB, AD therapeutics require the ability to circumvent the BBB, which is relatively difficult, but has been accomplished by various drugs that overcome the BBB and target the CNS [121].

## 5. Immunotherapy

**Figure 2:**
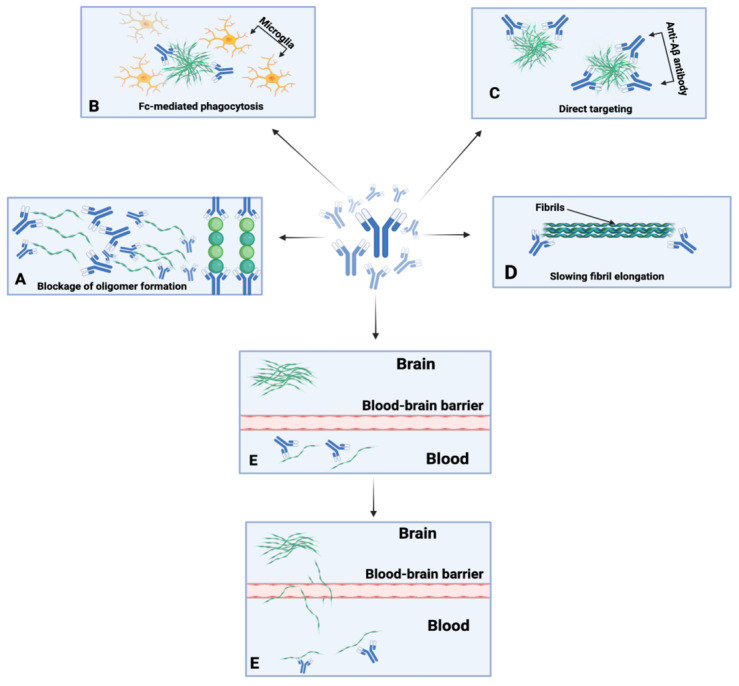
Possible mechanisms of anti-Aβ antibodies. Passive immunotherapies directly supply antibodies to the host and anti-Aβ antibodies interfere with the β-amyloid cascade at various levels. (**A**) Blocking of oligomer formation. (**B**) Plaque phagocytosis by microglia (Fc-mediated phagocytosis). (**C**) Direct mechanism of action towards Aβ plaques via antibody-mediated disassembly. (**D**) Slowing and halting of fibril elongation. (**E**) The refuted peripheral sink mechanism (monomer efflux from CNS) and shift in concentration gradient.

Immunotherapy refers to the field of immunology that localizes treatments via the induction, suppression, or enhancement of an immune response [122]. These treatments include vaccines, antibodies, cytokines, and cell-based therapies using immune cells [123]. Subsequently, there is active immunotherapy, which aims to stimulate or enhance the immune system of the host, and passive immunotherapy, which encompass therapies that directly supply antibodies to the host (Figure 2) [124]. Immunotherapy originated from the field of bacteriology when W. Busch and F. Fehleisen noted tumor regression in cancer patients after accidental infection with erysipelas [125]. Busch then became the first physician to inoculate a cancer patient with erysipelas in 1868, noting shrinkage of the malignancy in the patient [125]. Nonetheless, despite Busch’s and Fehleisen’s earlier contributions to the field, W.B. Coley is commonly referred to as the “father of immunotherapy” due to his treatment of soft tissue and bone sarcoma with Coley’s toxin, which encompasses a mixture of heat-killed *Serratia marcescens* and streptococcal organisms [125].

Numerous mechanisms of action have been hypothesized for anti-Aβ antibodies. These mechanisms of action encompass microglial plaque phagocytosis, Fc-receptor-mediated phagocytosis, β-amyloid toxicity neutralization, allosteric effects, and the monomeric efflux of Aβ from the CNS to circulation, among others (Figure 2) [118]. These varying mechanisms could be expected to become more prominent depending on the isotype, concentration, stage of the amyloid deposition process, and epitope specificity of the anti-Aβ antibodies. However, certain mechanisms have been refuted, such as the peripheral sink hypothesis. With regard to the peripheral sink hypothesis, Georgievska et al. demonstrated that inhibition of Aβ formation by BACE1 inhibitors is necessary and that the decline of Aβ in the periphery is not adequate to reduce Aβ levels in the brain [126].

Other than active and passive immunotherapies, some other therapeutics have been made to harness the immune system in AD. The targeting of immune molecules was consistent with earlier epidemiological research studies that proposed that long-term usage of anti-inflammatory medications were associated with a minimized risk of developing AD [127,128]. In turn, numerous clinical trials were designed with the intent of suppressing general inflammation. These trials included low dosages of prednisone, non-steroidal anti-inflammatory drugs, NF-κB blockers, statins, and more [129,130]. Nonetheless, no clear clinical benefits have been elucidated in AD patients. Furthermore, intravenous immunoglobulins have also been utilized clinically for the treatment of numerous infectious and autoimmune diseases and has demonstrated protective effects in animal models of AD [131,132]. However, intravenous immunoglobulin clinical trials have not yet demonstrated a positive effect [133,134,135]. Consequently, TNF-α is a pro-inflammatory cytokine that is deeply involved in AD and numerous peripheral inflammatory diseases. Peripheral neutralization of TNF-α was shown to be effective at reducing plaques of Aβ and neuronal dysfunction in 5xFAD mice [136]. Furthermore, a 41-subject, double-blind, placebo-controlled phase 2 trial administered Etanercept, which is a TNF-α decoy receptor with a well-tolerated response in the subjects [137]. Nonetheless, clinical trials with larger patient cohorts are necessary to further analyze the efficacy of this treatment strategy.

There have also been anti-aging strategies proposed to halt the aging-related clock of systemic decline. In terms of anti-aging strategies, most studies utilized rodents, but the reporting of molecules with rejuvenating capacity seem to be promising [46]. From 2014–2017, the phase I Plasma for Alzheimer’s Symptom Amelioration (PLASMA) examined the tolerability, feasibility, and safety of the infusion of blood plasma from younger donors to AD patients with mild-to-moderate cases [138]. The trial reported no serious adverse events and concluded that the treatment was well-tolerated, feasible, and safe [138]. However, the trial was limited due to the short duration and small sample size of the study. Consequently, in animal models, senolytics have recently appeared as novel anti-aging agents via the targeted destruction of senescent cells [139]. Thus, as these anti-aging strategies continue to be developed and employed in larger clinical trials, there could be some promising novel therapeutics for AD.

### 5.1. Active Immunotherapies

Active immunotherapies involve the administration of an adjuvant which induces an immune response for the production of antibodies [140]. The first study of active Aβ immunotherapy was conducted in 1999 utilizing the PDAPP mouse model where full-length human Aβ peptide and adjuvant were injected into young PDAPP mice and older PDAPP mice [75]. In the young PDAPP mice, the immunization generated Aβ antibodies which prevented plaque formation and neuritic dystrophy completely and in the older PDAPP mice, the extent of the amyloid deposits decreased significantly [75]. Following the model presented in the PDAPP mice, AN-1792 was the first human Aβ active immunotherapy and was formulated with synthetic full length human Aβ42 and an adjuvant [118]. The QS21 adjuvant was utilized as it favored the T_H_1 polarization of the T-cell response and enhanced antibody responses [141]. This study consisted of 372 patients who experienced mild-to-moderate AD; during phase I, patients were injected at an interval of day 0, and then again at weeks 4, 12, and 24 [142]. The conclusion of phase 1 trials demonstrated that the vaccine elicited an antibody response to Aβ42 and cleared plaque in the brain from post-mortem examinations. In phase II trials, AN1792 was injected at 1, 3, 6, 9, and 12 months; however, clinical trials were halted due to patients developing meningoencephalitis which were attributed to the T-cell response to Aβ [76]. Despite AN-1792 clinical trials having adverse effects, the results from this study encouraged the development of new methods of active anti-Aβ immunotherapy. Currently, CAD106, ABVac40, ACI-24, and UB-311 are the active anti-Aβ vaccines in phase II trials. Other active anti-Aβ immunotherapies include the peptide vaccine V950, Vanutide cridificar (ACC-001), and Lu AF20513 (Table 1) [140].

The CAD106 vaccine is developed by Novartis and works by inducing antibody production to reduce beta-amyloid plaques [143]. The vaccine is composed of shortened beta-amyloid fragments (Aβ1-6) to avoid Aβ T-cell activation [143,144]. Moreover, there were no signs of meningoencephalitis during the 52 weeks of phase I trial and the vaccine is undergoing safety trials while measuring the antibody response of the vaccine against beta-amyloid plaques during phase II trials [140].

The ABvac40 vaccine is developed by Araclon Biotech and is structured to target the C terminus of Aβ40 [145]. The vaccine is composed of short C-terminal fragments of Aβ40 and an aluminum hydroxide adjuvant. In order to generate an immune response against Aβ, the short repeats of the Aβ40 fragments are conjugated to a keyhole limpet cyanine (KHL) protein [146]. During phase I trials, patients with mild-to-moderate AD were enrolled, measuring the safety, immunogenicity, and tolerability of the vaccine [146]. As of now, ABvac40 has entered phase II trials where the safety and immune responses are being measured and this study is expected to conclude in December 2022 [146].

The ACI-24 vaccine is developed by AC Immune and contains the Aβ1-15 epitopes, excluding T-cell epitopes to avoid T-cell responses such as the ones from AN1792 [118,147]. The vaccine is liposome-based and induces β-sheet conformation-specific antibodies through the conformational epitopes of the liposomes [147]. During the preclinical trials with transgenic mice, there was success in the improvement in cognition and reduction in Aβ [148]. Currently, ACI-24 is in phase II trials to test the safety, immunogenicity, and tolerability of injections in patients with mild AD [148].

The UB-311 vaccine is developed by United Biomedical and contains Aβ1-14 and linked to a helper T-cell peptide epitope [148]. The mixture of peptides would induce B-cell responses while avoiding inflammatory responses from T-cells [148,149]. During phase I trials, Aβ antibody responses were generated in mild-to-moderate AD patients while the vaccine was safe and tolerable [149]. During the phase II trials, the primary goals were to collect further data on safety and immunogenicity [148].

Potential active Aβ-immunotherapies that were being studied but were terminated include peptide vaccine V950, ACC-001, and LU AF20513 [148]. The peptide vaccine V950 is composed of Aβ1-14 conjugated to ISCOMATRIX [148,150]. However, during trials, the study was terminated and there has not been any clinical data presented [148]. The vaccine ACC-001 is composed of Aβ1-7 and is conjugated with a non-toxic form of diphtheria toxin utilizing the QS-21 adjuvant [151]. During the phase II trials, it was found that the vaccine was safe and well-tolerated by patients. However, there were no improvements seen within patients resulting in the termination of the study [151]. The LU AF20513 vaccine is composed of repeats of Aβ1-12 while utilizing tetanus toxin [152]. The goal of this mixture was to avoid T-cell autoimmune response while activating B cell polyclonal antibodies [118,152]. During the phase I trial, the antibody titers, safety, and tolerability of the vaccine was measured. However, the study was terminated due to data on the efficacy of the vaccine [152]. 

**Table 1:** Overview of active Aβ immunotherapy vaccines.

Drug Name	Epitope	Mechanism of Action	Composition	Clinical Effects
AN1792 [76]	Aβ42	Reducing the formation of Aβ plaques	Full length human Aβ42 and QS21 adjuvant	Unsafe immune T-cell response resulting in meningoencephalitis
CAD106 [144]	Aβ1-6	Inhibits Aβ aggregation	Shortened beta-amyloid fragments	Safe, immunogenic but no reported clinical efficacy
ABVac40 [145,153]	Aβ33-40	Target the C-terminus of Aβ40	Aβ33-40, KHL, and alum hydroxide adjuvant	Safe, immunogenic, clinical results study to conclude in February 2022
ACI24 [147,154]	Aβ1-16	Inhibit the formation of Aβ plaques	Liposome based	Ongoing trial to measure safety, immunogenicity and tolerability
UB-311 [155,156]	Aβ1-14	Induce Aβ antibody response	Aβ1-14 and linked to a helper T-cell peptide epitope	Safe, immunogenic, and tolerable; but efficacy data are not published
Peptide vaccine V950 [150,157]	Aβ	Produce Aβ antibodies	Aβ1-14 conjugated to ISCOMATRIX	Terminated study with no clinical data
ACC-001 [158,159]	Aβ1-7	Reducing the formation of Aβ plaques	Aβ1-7, non-toxic form of diphtheria toxin, QS-21 adjuvant	Trial terminated due to no improvement in cognition
LU AF20513 [152,160]	Aβ1-12	Activate B cell polyclonal antibodies	Aβ1-12 and tetanus toxin	Terminated due to efficacy of vaccine

### 5.2. Passive Immunotherapies

Due to the amyloid hypothesis stating that plaque formation via amyloid aggregation is an instigative factor, various research studies have been conducted to determine which definitive isoform of Aβ engages in the amyloid cascade [118]. In passive immunotherapy, antibodies are generated ex vivo and directly injected into the patient [161]. This method is beneficial as specific epitopes can be targeted and, with repeated dosing, it results in the formation of anti-antibodies with potential neutralizing effects [118]. Passive immunotherapy against AD was first demonstrated in PDAPP mice models that received weekly intraperitoneal injections of an N-terminal-specific monoclonal antibody (mAb) against Aβ and 3D6 mAb [124]. Over the course of 6 months, it was found that the mice had reduced Aβ levels with an increase in Fc-receptor-mediated phagocytosis of Aβ [162]. This led to the creation of several anti-Aβ antibodies including bapineuzumab, gantenerumab, crenezumab, solanezumab, aducanumab, and BAN 20401 (Table 2).

Bapineuzumab was the first humanized form of the N-terminal-specific mAb to target Aβ plaques and induce Fc-receptor-mediated phagocytosis [163]. During phase I and II trials, the results indicated that there were cognitive benefits to patients who did not carry the APOE ε4 gene, and the overall safety and tolerance were established [164]. This prompted bapineuzumab to move into the phase III trial. However, there were no significant treatment effects for cognition and signs of amyloid-related imaging abnormalities (ARIA) were observed resulting in the termination of the study [148,164].

Gantenerumab is human immunoglobin antibody, binding with subnanomolar affinity to Aβ conformations in the N-terminal [148]. This antibody has been shown to reduce Aβ levels through the mechanism of inducing phagocytosis after binding to plaques [165]. During phase 1 trials, the antibody was safe and tolerable though there were some incidences of ARIA [166]. Further studies into phase II and phase III were conducted, but there were no significant differences in efficacy between the primary and secondary measures deeming no clinical benefit overall [118,148].

Crenezumab is a humanized anti-Aβ IgG monoclonal antibody that inhibits aggregation of Aβ in the monomer, oligomer, and fibrillar forms while also assisting in the process of disaggregation [167]. During phase 1 trials, the antibody presented adequate safety with no adverse effects, such as vasogenic edema or cerebral microhemorrhage [168,169]. Based on this conclusion, phase II clinical trials proceeded utilizing a higher concentration of the antibody that was injected intravenously every 4 weeks for a total of 73 weeks [170]. An increase in cerebrospinal fluid β-amyloid_1-42_ was observed suggesting the targeted engagement in the brain as achieved. However, there was no change in cognition [170]. An ultra-sensitive immunoassay conducted on the cerebrospinal fluid (CSF) samples from patients for oligomeric Aβ found that over 85% of the patients had reduced levels of oligomeric Aβ in CSF [170]. This suggests that the treatment reached the intended target in the brain [171].

Solanezumab is another humanized monoclonal IgG1 antibody that targets the mid-domain of soluble Aβ [172]. During early clinical trials, there were no improvements on patient’s cognition in moderate AD. However, in patients with mild AD, there was a reduction in the rate of decline [173]. In 2020, the trial for phase III which started in 2013 had failed to provide any statistically significant results during the therapy [174].

Aducanumab is a human IgG1 monoclonal antibody that binds to aggregated Aβ [175]. During phase I, a single intravenous infusion was given to patients with mild-to-moderate AD to determine the safety. During the next phase, 1, 3, 6, and 10 mg/kg doses were given to determine the effects on mild AD [148,176]. This phase revealed that there was a statistically significant slowing of clinical decline in all doses. The phase III study of aducanumab was halted in March 2019 due to the lack of meeting primary goals [148]. However, in October 2019, the FDA approval process for aducanumab began again [177,178]. In June 2021, aducanumab was approved for medical use by the FDA but there was controversy surrounding this decision as conflicting results were evaluated over the effectiveness of the treatment [148]. The normal route of standard approval was not granted, but the FDA opted for accelerated approval [179]. The criticism drawn from this decision related to whether or not there were any cognitive benefits derived from aducanumab or if it will only bring false hope and harm the patient, as it will not actually halt the progression of the disease. Currently, they are waiting for the post marketing trial ending in 2030 to prove that the drug has cognitive benefits [179]

BAN2401 is the humanized version of the mouse mAb158 and targets large, soluble Aβ protofibrils [180]. During the phase 1 trial, the safety and tolerability was tested and there were no reported ARIA in patients with mild-to-moderate AD [180]. The dosing regimen was 10 mg/kg every two weeks for four months and the results indicated that the antibody entered the CSF but the efficacy was unknown [180]. Phase II trials began to test different intravenous doses to determine the 12-month baseline of cognitive tests and safety [118,180]. It was announced in 2017 that there were no cognitive benefits during the 12-month time period. However, the protocol for the trial was changed in February 2018 which offered up to 5 years of treatment [181]. Currently, a large 4 year BAN2401 study is being conducted to determine the changes in beta-amyloid and cognitive functions and this trial will run until October 2027 [182]. 

**Table 2:** Overview of passive Aβ immunotherapies.

Drug Name	Mechanism of Action	Clinical Effects
Bapineuzumab [183,184]	Target Aβ plaques and induce Fc-receptor-mediated phagocytosis	No clinical benefit and ARIA was observed resulting in termination
Gantenerumab [165,185]	Inhibits formation of Aβ plaques	No clinical benefit
Crenezumab [168]	Inhibits Aβ aggregation and assists with disaggregation	Ongoing trial determining efficacy of treatment
Solanezumab [186,187]	Targets the mid-domain of soluble Aβ	No clinical efficacy reported
Aducanumab [178,188]	Reduce Aβ oligomers	Phase III trials terminated due to futility analysis (no cognitive benefits), received approval for medical use by FDA
BAN2401 [182]	Reduces large, soluble Aβ protofibrils	Safe, tolerable, and unknown efficacy trial will run until October 2027

## 6. Future Direction of AD Immunotherapy

Immunotherapy should ideally be viewed as a type of precision and personalized medicine. In terms of personalized medicine, the general population has an adopted immunity derived from the parents, meaning that the degree of gene inheritance from parents is varied, and the immune response elicited by the treatment could potentially vary person-to-person [189]. Thus, prescribing identical treatments to different hosts could potentially elicit aberrant responses in distinct subjects. Due to this, prescribing individuals different dosages of treatments could be crucial to ideally eliciting similar responses [190]. Furthermore, this can also lead to grouping subjects in similar groups based on their dosage and immune profile, with the expectation that the groups will elicit a more homogeneous response, which is precision medicine [191].

In order to ensure more progressive advances in AD immunotherapy, it is crucial to consider that AD substantiates as a heterogeneous multifactorial disorder with a plethora of pathobiological and clinical subtypes [4,5,7]. Although there are major subtypes categorized on brain atrophy and tau pathology dissemination—such as minimal atrophy, typical, limbic predominant, and hippocampal sparing—there are also a plethora of differing variants, such as mild dementia, cortical atrophy, corticobasal syndromal, primary progressive aphasia, amyloid positive, and immunosenescent variants [4]. Even more, its polygenic and multifactorial nature lead to various defective genes apportioned through the human genome that may be conducive in the pathogenesis, resulting in neurofibrillary tangles and amyloid deposition [5]. Furthering the complexities associated with AD, there are also pathological and clinical intertwines with differing pathologies such as cerebrovascular and Lewy Body disease [4,192]. Due to this, the likelihood of developing a universal therapeutic agent for AD is slim. Instead, AD immunotherapy should consist of prescreening for the explicit therapeutic to reach the treatment threshold [121]. As such, AD immunotherapy should be focused on precision and personalized medicine [110].

In recent times, there has been momentous progress in the treatment of some monogenetic disorders, such as cystic fibrosis and various cancers, via the utilization of precision medicine [110]. However, for other diseases such as AD, precision medicine is still in the initial stages. Currently, most therapeutic and preventative measures for AD are largely ineffective, with the presently utilized therapies centralizing on cholinesterase inhibitor activity, encompassing donepezil, rivastigmine, and galantamine, or suppressing ionotropic glutamatergic signals by memantine [193,194]. However, all of these medications are only administrated consequent to the onset of symptoms, and have sleep disturbance, nausea, and diarrhea as their frequent side effects [110].

The fundamental objective of personalized and precision medicine is to allow physicians and researchers to efficiently and precisely determine the most efficacious therapeutic or preventative treatment for a patient based on their risk profile [195]. In order to do this, clinicians routinely utilize information-technology and clinical tests that are economically viable [110]. This would aid in the extrication of the biological intricacy underlying the patient’s disease. Furthermore, there are a plethora of prerequisites that must be considered in order to develop a safe and efficacious treatment for AD; such as targeting of at least one of the major pathological proteins, Tau or Aβ. Furthermore, the impaired immune system of geriatric subjects should be considered, and balance should be brought to the acquired and innate immune systems. Consequently, avoidance of repeated treatment if possible—while causing no harm to the patient—would be beneficial. This would allow for the modulation of the AD patient’s immune system to effectively treat AD [121].

There has recently been a significant advancement in imaging and genomic tools and procedures, with notable advances in the identification of underlying genetic risk variants precisely specifying molecular pathways [110,196]. Even more, there has also been a progression in the detection technology utilized for pathophysiological processes [197]. This has led to the incorporation of precision and personalized medicine in a variety of clinical trials—including the Anti-Amyloid Treatment in Asymptomatic Alzheimer Disease, the Alzheimer’s Prevention Initiative, and the Dominantly Inherited Alzheimer Network Trial—with their focal point being patients with recognized AD risk factors, as well as neuroimaging and biofluid biomarkers to aid in the detection of AD onset [198,199,200]. Even though the prosperous and advantageous application of personalized and precision medicine in AD requires a considerable amount of work to appropriately recognize risk factors, AD subgroups, and pathological processes, it is hoped that this application will aid in the production of novel interventions that will advise clinical practice.

## Data Availability

Not applicable.
